# Hemophagocytic Lymphohistiocytosis Complicating a Case of Drug-Induced Liver Injury Precipitated by Cephalexin: A Rare Consequence of Commonly Prescribed Medications

**DOI:** 10.1155/crhe/4600879

**Published:** 2025-05-19

**Authors:** Matthew T. Newman, Thu Anne Mai, Joe McClanaghan, Nicholas Burley, Tamira Robinson, Yang Jiang, Amandeep Sahota

**Affiliations:** ^1^Department of Internal Medicine, Kaiser Permanente Los Angeles Medical Center, Los Angeles, California, USA; ^2^Department of Gastroenterology, Kaiser Permanente Los Angeles Medical Center, Los Angeles, California, USA; ^3^Department of Hematology and Medical Oncology, UCLA Medical Center Olive View, Sylmar, Los Angeles, California, USA; ^4^Department of Transplant Hepatology, Kaiser Permanente Los Angeles Medical Center, Los Angeles, California, USA; ^5^Department of Hematology and Medical Oncology, Kaiser Permanente Los Angeles Medical Center, Los Angeles, California, USA

## Abstract

Drug-induced liver injury (DILI) is a relatively rare clinical syndrome that accounts for a significant proportion of acute liver failure progressing to transplant in the United States. Some drugs such as acetaminophen are classically associated with a predictable pattern of DILI that can often be reversed with prompt administration of guideline-directed therapeutics. In other cases, commonly prescribed medications can lead to an unpredictable variant of DILI in certain vulnerable populations for which few guidelines on management exist, likely in part due to the heterogeneity of precipitating toxins. We report a case of idiosyncratic DILI caused by cephalexin that progressed to fulminant hemophagocytic lymphohistiocytosis (HLH) in a young and previously healthy patient, alongside our experiences with therapeutic management and outcomes guided by a multidisciplinary team.

## 1. Introduction

Drug-induced liver injury (DILI) is an infrequently reported subtype of acute liver injury, affecting roughly 14/100,000 individuals annually [1]. Despite its uncommon presentation, DILI cases make up over 50% of all cases of acute liver failure in the United States, making it a highly relevant topic in the setting of expanding usage of both prescription medications and herbal/dietary supplements among the general population, as well as the implication of commonly prescribed medications (such as antibiotics) in many cases thus far described [2, 3]. Hemophagocytic lymphohistiocytosis (HLH) is a rare syndrome characterized by the release of a deleterious inflammatory milieu by dysregulated immune cells leading to systemic end-organ damage, which often occurs in the setting of an underlying immune disturbance. Herein, we detail a case of DILI in a young patient treated with cephalexin complicated by secondary HLH alongside their treatment course and clinical outcomes, in the hopes of bringing attention to this potentially devastating complication alongside a summary of available therapeutic strategies.

## 2. Case Presentation

A 24-year-old male with no significant past medical history initially presented to urgent care with chief complaints of nausea, vomiting, and body aches of five days duration. Prior to this, the patient had completed a limited course of antibiotics for superficial cellulitis of the right wrist, which had been diagnosed the previous week following a spider bite. Culture data was not obtained, and due to the limited nature of the infection he was empirically treated with oral cephalexin, 500 mg four times daily for 7 days.

On initial presentation, the patient was ill-appearing though afebrile and hemodynamically stable. His physical exam was notable for diffuse jaundice and scleral icterus, alongside tenderness to moderate palpation over the epigastric region and right upper quadrant (RUQ) of the abdomen. Initial laboratory studies showed only a mild normocytic anemia and an unremarkable electrolyte panel including intact renal function. Ancillary testing revealed a significant transaminitis in the thousands in a mixed hepatocellular and cholestatic pattern. The patient was then transferred to a higher level of care at the San Bernadino Kaiser Permanente Medical Center for continued management and workup. Further testing here revealed an elevated INR to 1.4 and decreased albumin to 3.0 g/dL, which alongside the hepatic injury was concerning for synthetic liver dysfunction and acute liver failure. Acetaminophen levels were undetectable. A computed tomography (CT) scan of the abdomen and pelvis revealed normal size and attenuation of the liver parenchyma alongside periportal edema and gallbladder wall thickening without obstructive cholangiopathy.

**Figure 1:**
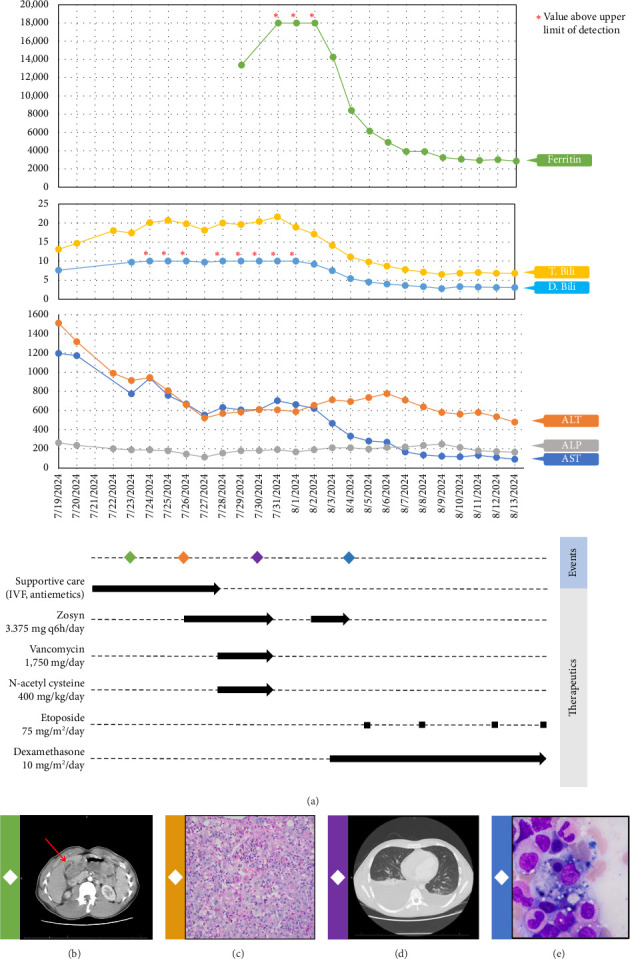
(a) Time course of attempted therapeutic interventions and tracking of relevant lab values and significant events. (b) CT scan highlighting sterile cholecystitis. (c) Liver biopsy performed showing inflammation with parenchymal damage, consistent with DILI. (d) Patient develops new fevers with repeat CT scan showing bilateral pleural effusions, hepatosplenomegaly. (e) Bone marrow biopsy performed showing hemophagocytosis.

Available data at the time suggested an underlying diagnosis of DILI with the suspected precipitant of cephalexin in the absence of any other identifiable toxin (such as acetaminophen) or immediately obvious infectious agent. Additional workup and therapeutic interventions for our patient's care proceeded as outlined in Figure 1 and Table 1. After being diagnosed with DILI, without available evidence-based therapeutics to mitigate hepatic damage, the patient was placed on supportive measures including intravenous fluids and anti-emetics. His transaminitis began to improve, but his direct hyperbilirubinemia continued to worsen. After review with general surgery consultants, the patient was diagnosed with acute cholecystitis and was taken back for an urgent laparoscopic cholecystectomy which was well tolerated. Despite this, the patient's hyperbilirubinemia continued to worsen. Concern for an occult infection grew, and the patient was placed on broad spectrum antibiotics with piperacillin/tazobactam (Zosyn) and vancomycin; though notably never became culture positive and was without signs of systemic inflammatory response syndrome (SIRS). Liver biopsy was formally undertaken, which returned with inflammation and parenchymal damage consistent with DILI, shown in Figure 1(c). Rheumatologic workup revealed an elevated anti-nuclear antibody (ANA) titer (1:320), though in the absence of elevated anti-smooth muscle or anti-mitochondrial antibodies, auto-immune contributors were favored to be less likely (Table 1).

A brief trial of N-acetyl cysteine (NAC) and continued antibiotic coverage failed to alleviate the direct hyperbilirubinemia, and on hospital day 10 the patient began to experience fevers and new thrombocytopenia. Repeat CT imaging revealed bilateral pleural effusions and new hepatosplenomegaly without an obvious infectious nidus. Pulmonary consultants performed a diagnostic thoracentesis, which proved exudative by Light's criteria without findings of infectious agents or malignant cells on culture or microscopy respectively. Hematology consultation was placed to further differentiate the thrombocytopenia, and included HLH in the differential diagnosis. Ancillary testing revealed a markedly elevated ferritin, and along with a subsequent bone marrow biopsy showing hemophagocytosis, the clinical suspicion for HLH was high enough to start guideline-directed therapeutics including dexamethasone and etoposide (Figure 1(a)). Ultimately, the patient was found to meet six out of eight necessary diagnostic criteria for HLH (Table 2). Direct hyperbilirubinemia and ferritin were continuously tracked and began to fall alongside symptomatic improvement. Following 2 weeks of additional therapy, the patient was discharged on hospital day 26 with appropriate follow up scheduled in the outpatient setting. At this time, the patient remains well and is currently nearing completion of his treatment for HLH.

## 3. Discussion

The clinical course of DILI typically proceeds along one of two distinct pathophysiological pathways. Intrinsic DILI functions in a dose-dependent fashion and manifests following exposure to common medications (including acetaminophen, valproate, statins, etc.) that act on the molecular level to interfere with mitochondrial functioning and metabolism, as well as through the creation and accrual of reactive oxygen species [1, 4]. Intrinsic DILI is both predictable and reproducible, and has an expected latency period of hours to days from exposure to onset of hepatocellular injury in most patients [1, 4, 5]. Conversely, idiosyncratic DILI does not track a predictable course following drug exposure and is without clear dose-dependence or expected latency periods, and is thought to be perpetrated through an overwhelming adaptive immune response in genetically vulnerable individuals [4]. Isolation of metabolite-primed T cells from peripheral blood and the identification of several human leukocyte antigen (HLA) subtypes through genome wide association studies (GWAS) in patients with DILI support the idea of genetic substrates for idiosyncratic DILI, though more studies are needed to fully elucidate what germline and environmental factors contribute to this phenomenon [6–8]. Current mechanistic hypotheses underlying predisposition to hepatic injury in such individuals include aberrant T cell activation by drug-protein complexes presented by certain HLA molecules, as well as gene polymorphisms affecting innate immune cell function and protection against oxidative stress [8]. Common culprit medications leading to idiosyncratic injury identified by a landmark study of DILI in the United States included antibiotics (45.5%) and central nervous system agents (15%), with amoxicillin-clavulanate being the single most commonly implicated agent [9]. Only one case of DILI attributable to cephalexin was captured in this study of 300 cases, and a recently published database-driven study of over 300,000 patients found fewer than 20 cases of cephalexin-mediated DILI, making this an exceeding rare phenomenon [10]. It is highly possible that our patient harbored an underlying genetic susceptibility or HLA subtype that predisposed him to develop this reaction, and baseline HLA-typing could be considered in his case to better guide future antibiotic selection for the patient and relatives, though was not undertaken at the time of his presentation. While unifying guidelines on antibiotic stewardship related to DILI are not available in part due to heterogeneity of suspected mechanisms, drug preference in those with suspected predisposition to such reactions should be guided by known drug cross-reactivity among beta lactams and appropriate selection of alternative agents with similar bacterial coverage [11]. Unfortunately, few evidence-based approaches exist for counteracting the deleterious effects of DILI, with most guidelines focusing on withdrawal of the causal agent. Specific therapies such as NAC and L-carnitine are efficacious in instances of acetaminophen and valproate toxicity respectively, and while limited data exists highlighting a survival benefit of NAC in DILI not otherwise caused by acetaminophen, additional larger studies are needed before these findings can be generalized [12–14].

HLH is a complex syndrome of excessive inflammation related to dysfunctional cytokine release by immune cells [15, 16]. Primary, or inherited, HLH is associated with underlying genetic mutations that lead to aberrant baseline immune cell activity via impaired cellular signaling and trafficking of cytotoxic cargo in granulocytes [15–17]. Secondary HLH occurs in those without known germline genetic mutations in which an overwhelming pro-inflammatory state is triggered as a consequence of a principal immune disturbance, such as systemic infection, malignancy, and autoimmune disease [15, 16]. A recent epidemiological investigation of cases of HLH in the United States determined that malignancies (namely heme malignancies) are the causal precipitant in nearly one-third of cases (30.7%), followed by infection (24.3%) and autoimmune disease (20.8%) [18]. Infectious agents are typically viral (such as Ebstein Barr Virus [EBV], Cytomegalovirus [CMV]) though can also be bacterial or fungal in certain hosts [18]. No findings in our patient's history, laboratory studies, or imaging were congruent with malignancy, and he was thoroughly vetted to ensure that no associated infection was missed; by way of culture, antigen/antibody testing, as well as through a send-out liquid biopsy probing for cell-free DNA (Karius) [19]. Further, genetic testing for primary HLH was undertaken in his case and did not identify the presence of any deleterious mutations within known loci [16]. Macrophage activation syndrome (MAS) is a subset of secondary HLH that develops in those with autoimmune disease, commonly in patients with systemic juvenile idiopathic arthritis (sJIA) [15]. The significance of his positive ANA titer was discussed with rheumatology consultants, and given that all further recommended rheumatologic antibody testing was negative and that the patient lacked clinical diagnostic criteria necessary for sJIA or other autoimmune conditions, there was a low suspicion for contributory autoimmune disease. Diagnostic criteria (outlined in Table 2) and therapeutic approach for HLH are derived from existing international studies HLH-2004 and its predecessor HLH-94 [20]. Upfront administration of concurrent dexamethasone and etoposide for 2 weeks is standard of care, followed by administration of cyclosporine A for further control typically at week 9 (which our patient received in the outpatient setting) and optional intrathecal methotrexate if neurologic involvement is suspected [20].

Our clinical opinion, informed by multiple subspecialty experts, was that the primary insult of idiosyncratic DILI related to cephalexin created the necessary inflammatory substrate to further dysregulate the patient's immune system to trigger the development of secondary HLH. The onset of fever, cytopenia, and pleural effusion in our patient likely represented the onset of HLH, the latter of which has been shown to be a marker of severe disease and an independent negative prognostic factor for overall survival [21]. Scant literature has been published on cases of DILI leading to secondary HLH, which on our review has only been captured in two cases seen with allopurinol and lamotrigine toxicity [22, 23]. Despite the rarity of both of these conditions, prompt recognition of DILI and HLH are essential to avoid progression to liver transplant as well as fatal complications, which in our case was avoided thanks to expert interdisciplinary opinion and collaboration which have resulted in the sustained well-being of our patient. To better predict vulnerable populations to these toxicities, additional initiatives to better correlate susceptibilities to specific agents associated with DILI are necessary, which we hope will be seen alongside increased awareness in the literature and expansion of existing toxicity-reporting infrastructure [24].

## Figures and Tables

**Table 1:** Ancillary laboratory testing detailing holistic workup for underlying illness.

Test	Value
Hepatitis viral panel	Negative
CMV	Negative
EBV	Negative
Microbial cell-free DNA testing	Negative
Anti-nuclear antibody	1:320
Anti-smooth muscle antibody titer	< 1:20
Anti-mitochondrial antibody titer	< 1:20
Alpha 1 anti-trypsin	180 mg/dL
Ceruloplasmin	29 mg/dL
Triglycerides	315 mg/dL
Ferritin	13,389 mg/dL
IL-2 receptor levels	15,746 pg/mL

**Table 2:** Diagnostic criteria for HLH and those met during course of hospitalization.

HLH diagnostic criteria (at least 5/8)	
Criterion	Met?
Fever	✓
Splenomegaly	✓
Cytopenia involving ≥ 2 cell lineages	X
Hemophagocytosis	✓
Hypertriglyceridemia and/or hypofibrinogenemia	✓
Serum ferritin ≥ 500 ng/mL	✓
Elevated IL-2 receptor levels	✓
Low or absent natural killer (NK) cell activity	Was not obtained

## Data Availability

The data that support the findings of this study are available on request from the corresponding author. The data are not publicly available due to privacy or ethical restrictions.
